# Fourier-transform infrared spectroscopy with machine learning for rapid O-serogrouping and typhi differentiation of clinical *Salmonella* isolates

**DOI:** 10.3389/fmicb.2026.1847592

**Published:** 2026-07-15

**Authors:** Mohammed Suleiman, Princess Morales, Elizabeth Ilagan, Miriam Cordovana, Rafal Pfeiffer, Andrés Pérez-López

**Affiliations:** 1 Department of Pathology, Sidra Medicine, Doha, Qatar; 2 Department of Biomedical Sciences, Qatar University, Doha, Qatar; 3 Bruker Daltonics GmbH & Co. KG, Bremen, Germany; 4 Department of Pathology and Laboratory Medicine, Weill Cornell Medicine in Qatar, Doha, Qatar

**Keywords:** FTIR, IR biotyper^®^, *Salmonella*, *Salmonella typhi*, serotyping

## Abstract

*Salmonella enterica* remains a leading cause of gastrointestinal infections worldwide, requiring rapid and accurate typing for effective outbreak management and clinical treatment. Traditional serological methods, such as the Kauffmann–White scheme, are labor-intensive, time-consuming, and require specialized expertise. This study evaluated the IR Biotyper (IRBT) system, which combines Fourier-transform infrared (FTIR) spectroscopy with machine learning algorithms, for rapid classification of clinical *Salmonella* isolates at the O-serogroup level and for differentiating *S*. Typhi from non-typhoidal *Salmonella* (NTS) in a routine pediatric microbiology setting in the Middle East. A total of 164 clinical isolates from blood and stool cultures were analyzed, generating 1,925 spectra for serogroup classification and 790 spectra for the Typhi vs. NTS classifier. Using the Wellcolex™ Color *Salmonella* kit as a reference, the IRBT O-group classifier achieved an overall accuracy of 98.2%, correctly identifying 161 of 164 isolates. The system reached 100% accuracy for Groups A (*n* = 4, 95% CI: 39.8%–100%), A+E/G (*n* = 5, 95% CI: 47.8%–100%), B (*n* = 40, 95% CI: 91.2%–100%), C+D (*n* = 13, 95% CI: 75.3%–100%), D (*n* = 49, 95% CI: 92.7%–100%), and isolates expressing the Vi antigen (*n* = 2, 95% CI: 15.8%–100%), while Group E/G showed lower accuracy at 89.5%. The Typhi vs. NTS classifier achieved 100% accuracy in both internal validation (*n* = 34, 95% CI: 89.8%–100%) and independent testing (*n* = 31, 95% CI: 88.8%–100%), demonstrating complete concordance with the Vi antiserum assay. These results indicate that FTIR spectroscopy offers a rapid, reliable, and user-friendly alternative to conventional serotyping, reducing turnaround time to under 2 h. Integration of this technology into clinical microbiology workflows provides a robust tool for real-time pathogen monitoring and enhanced public health response.

## Introduction

*Salmonella enterica* remains one of the major foodborne pathogens globally, representing a significant cause of gastrointestinal illness and systemic infections in humans. Typhoid fever is estimated to cause between 9.9 and 24.2 million cases and 75,000–208,000 deaths per year while non-typhoidal *Salmonella* (NTS) is estimated to account for over 500,000 cases of invasive disease and approximately 77, 500 deaths in 2017, imposing a substantial burden on healthcare systems worldwide (Antillón et al., 2017; Buckle et al., 2012; GBD 2017 Non-Typhoidal Salmonella Invasive Disease Collaborators, 2019). Transmission of *S. enterica* to humans occurs through the ingestion of wide variety of contaminated food products and has been implicated in both sporadic and outbreak-associated cases (Antunes et al., 2016; Zizza et al., 2024). For effective outbreak investigation, surveillance, and proper clinical management, discrimination of *S. enterica* at subspecies and serotype levels is essential. Conventional typing methods include serological serotyping, phage typing, and molecular DNA-based approaches (Grimont and Weill, 2007; Sabat et al., 2013; Tang et al., 2019; Wattiau et al., 2011). Among these methods, serological serotyping based on the Kauffmann–White scheme, which relies on agglutination reactions targeting the somatic (O) and flagellar (H) antigens, continues to be the most widely used phenotypic method internationally (Grimont and Weill, 2007; Wattiau et al., 2011). Although serological serotyping methods can differentiate the major serovars of *Salmonella*, they have well-recognized limitations: they are labor-intensive, time consuming, costly, often require high technical skill, and in many settings are restricted to reference laboratories (Sabat et al., 2013; Tang et al., 2019; Wattiau et al., 2011). The use of matrix-assisted laser desorption ionization-time of flight mass spectrometry (MALDI-TOF MS) in typing *Salmonella* is a promising tool, but it requires the incorporation of bioinformatic and machine learning which is not commonly accessible in many laboratories (Ren et al., 2025). Given these existing challenges in the available diagnostics for *Salmonella* typing, there is a clinical and epidemiological need for an alternative *Salmonella* typing method that is faster, simpler, accessible, and cost-effective in clinical and public health laboratories.

Fourier-transform infrared (FTIR) spectroscopy has recently emerged as a promising rapid, user-friendly, and cost effective technique for bacterial differentiation across various taxonomic levels, including species, serogroup, and strain (Al-Fraihat et al., 2025; Lasch and Naumann, 2015; Orlandi Barth et al., 2025; Quintelas et al., 2018). Its utility has been particularly demonstrated in high-throughput clinical settings for the real-time identification of multidrug-resistant lineages and the management of hospital outbreaks (Gonçalves et al., 2025; Novais et al., 2024; Rodrigues et al., 2020). The diversity of *S. enterica* surface antigens and the high variability in O-antigen polysaccharide structures make this species particularly suitable for FTIR-based differentiation. Several studies have demonstrated that FTIR spectroscopy can successfully discriminate *S. enterica* serogroups and serotypes when combined with advanced data analysis techniques such as multivariate statistical modeling and machine learning (Campos et al., 2018; Cordovana et al., 2021, 2022; Dopuğ et al., 2025; Fredes-García et al., 2025; Muchaamba and Stephan, 2024; Napoleoni et al., 2024). Building on this evidence, the present study was designed as an independent clinical validation of a commercially available FTIR-based method in a routine pediatric microbiology setting in the Middle East. We evaluated the system’s performance using a collection of clinical isolates from Qatar, focusing on automated O-serogroup classification and the differentiation of *S. Typhi*.

The study aimed to determine the accuracy and reliability of this FTIR-based approach as a rapid and cost-efficient alternative for *Salmonella* typing in our clinical microbiology laboratory at Sidra Medicine.

## Materials and methods

### Sample collection, culture media and conditions

A total of 164 well-characterized, non-duplicate *S. enterica* isolates of clinical origin were included in this study. These isolates were collected from stool and blood specimens between January 2019 and September 2025 at the Microbiology Laboratory of Sidra Medicine, a pediatric tertiary care hospital in the State of Qatar. The isolates were stored at −80 °C and subsequently retrieved on Columbia agar with 5% sheep blood (Oxoid Limited, Basingstoke, United Kingdom). Prior to IRBT analysis, subculture was performed on Columbia agar with 5% sheep blood and/or Hektoen Enteric agar (Oxoid Limited, Basingstoke, United Kingdom). Plates were incubated aerobically at 35 °C ± 2 °C for 24 h.

### Bacterial identification and serological testing

All isolates were identified at the genus level using matrix-assisted laser desorption/ionization time-of-flight mass spectrometry (MALDI-TOF MS) (). Serological serotyping was performed for all isolates using BD Difco Salmonella Vi Antiserum (Becton, Dickinson and Company, Maryland, United States) to identify *S. Typhi*, and the Wellcolex™ Color Salmonella kit (Remel Europe Limited, Kent, United Kingdom). The Wellcolex Salmonella kit detects strains belonging to serogroups A, B, C, D, E, or G, as well as strains expressing the Vi antigen (Wellcolex Colour Salmonella, 2016). Isolates that yielded discordant results between the Wellcolex™ kit and the FTIR-based approach, or those where the Wellcolex™ kit could not provide definitive discrimination (e.g., between Groups E and G), were referred to a reference laboratory for confirmatory serotyping using BD Difco reagents (Becton, Dickinson and Company, Maryland, United States). For the purposes of accuracy calculation, a gold standard truth was established for each discrepant case based on the reference laboratory’s findings. In instances where the reference laboratory provided a definitive serogroup that matched one of the two conflicting results, that method was treated as the truth. In cases where the reference laboratory was unable to type the isolate, the initial Wellcolex™ result was maintained as the reference.

All tests were performed according to the manufacturers’ instructions.

### Sample preparation for FTIR analysis

Fourier-transform infrared analysis was performed by the IR Biotyper^®^ system (IRBT - Bruker Daltonics, Germany). Sample preparation for IRBT analysis was performed according to the manufacturer’s instructions. Briefly, a 1-μL loopful of bacterial colonies collected from the confluent growth area of the culture plate was resuspended in an IRBT vial containing 50 μL of 70% ethanol and inert metal cylinders. After vortexing, 50 μL of deionized water was added, and the mixture was vortexed again. Subsequently, 15 μL of the bacterial suspension was pipetted onto a 96-spot silicon IRBT sample plate in four technical replicates. After spotting, the plate was dried at 35 °C ± 2 °C for 10 min. For quality control, Infrared Test Standards (IRTS 1 and IRTS 2) provided in the IRBT kit were resuspended in 100 μL of deionized water, followed by the addition of 60 μL of absolute ethanol and thorough mixing. A 10-μL aliquot of each suspension was then applied in duplicate onto the IRBT sample plate and allowed to dry. All clinical isolates were tested using two biological replicates derived from independent subcultures performed on two consecutive days. Each biological replicate was analyzed in four technical replicates, resulting in eight spectra per isolate per medium.

### Spectra acquisition and analysis

Spectra acquisition, automated processing, and analysis were performed by the IRBT software V4.0 (Bruker Daltonics). Spectra were acquired in transmission mode, in the mid-IR spectral range of 4,000–600 cm^–1^ using the IRBT spectrometer and the OPUS software version 8.2.28, applying the default settings recommended by the manufacturer. After spectra acquisition, the second derivative over nine datapoints was calculated, using the Savitzky-Golay algorithm. Spectra were then vector-normalized and interpolated to have one data point for each integer wavenumber value. The spectral region corresponding to the absorption of carbohydrates (1,300–800 cm^–1^) was used for the analysis. Prior to sample spectra acquisition, the quality control measurements (IRTS 1 and IRTS 2) were performed for each run. All 164 isolates were cultured on Columbia Blood Agar (1,312 potential spectra), and a subset of 81 isolates was additionally cultured on Hektoen Enteric Agar (648 potential spectra), totaling 1,960 potential spectra. Following automated quality control to exclude spectra with low signal-to-noise ratios or insufficient biomass, a final dataset of 1,925 spectra (98.2%) was retained for analysis. For the O:9 serogroup-specific classifier (*n* = 65), 34 isolates cultured on both media provided 544 spectra for model training, while the remaining 31 isolates provided an independent test set of 246 QC-passed spectra.

### Exploration method analysis

The discriminatory power of the IRBT in distinguishing different O-serotypes of *S. enterica* was investigated, followed up by the differentiation between *S.* Typhi and non-Typhi isolates within the O:9 group. Principal components analysis (PCA) and linear discriminant analysis (LDA) were applied to the whole dataset of the clinical isolates measured in our study (*n* = 164).

### Automated classifiers and machine learning algorithms

The first part of our evaluation involved determination of the “O” antigen using the ready-to-use Salmonella serogroup classifier available in the IRBT software (Salmonella O-groups classifier V3). This classifier recognizes 36 O-groups (O:1,3,19 [E4], O:11 [F], O:13 [G], O:16 [I], O:17 [J], O:18 [K], O:2 [A], O:21 [L], O:28 [M], O:3,10 [E1–E3], O:30 [N], O:35 [O], O:38 [P], O:4 [B], O:41 [S], O:42 [T], O:43 [U], O:44 [V], O:45 [W], O:47 [X], O:48 [Y], O:50 [Z], O:51, O:53, O:56, O:57, O:58, O:6,14 [H], O:60, O:61, O:65, O:66, O:7 [C1–C4], O:8 [C2–C3], O:9 [D1], and O:9,46 [D2]). Additionally, a group defined “Others” is also included, representing strains of different serogroups exhibiting the rough colony morphotype. The classifier was trained with 158 isolates representing the above-mentioned 36 O-serogroups, as well as the “Others” group.

In this study, the O-groups classifier was challenged using a set of 164 *Salmonella* isolates as an independent evaluation set. These isolates were completely independent from the 158 isolates utilized by the manufacturer to construct the training database.

The second part of our evaluation involved the development and validation of a secondary classifier to differentiate the O:9 serogroup into *S.* Typhi and non-Typhi *Salmonella* (NTS). To evaluate this classifier, 65 well-characterized O:9 isolates (29 *S.* Typhi and 36 NTS) were divided into a training set and an independent test set using a random split stratified by class to maintain class balance across both cohorts. A machine-learning predictive model (classifier) based on principal component analysis and artificial neural network (PCA–ANN) was constructed using the IRBT software V4.0. The training set (34 isolates; 544 spectra), derived from cultures on Columbia agar with 5% sheep blood and/or Hektoen Enteric agar, included 15 Typhi and 19 NTS isolates. This cohort was utilized for model construction and internal 4-fold cross-validation. During the model training, 100 cycles were performed to enable the algorithm to learn the distinguishing spectral features and to recognize consistent patterns, thereby supporting classification of unknown samples. The independent test set (31 isolates; 246 spectra), containing the remaining 14 Typhi and 17 NTS isolates, was utilized to validate the classifier’s performance. Performance results were automatically generated by the software, and overall accuracy was calculated using a confusion matrix.

### Ethics statement

This study was conducted on previously collected bacterial isolates that were available in our laboratory and originally derived from pediatric clinical samples. The use of these de-identified isolates did not require informed consent in accordance with institutional policies. The study protocol was reviewed and approved by the Institutional Review Board (IRB) at Sidra Medicine (1988373-5).

## Results

### Samples tested

A total of 164 clinical *Salmonella* isolates were analyzed in this study, comprising 27 isolates from blood cultures and 137 from stool cultures. Using the Wellcolex™ Color *Salmonella* assay, serogrouping of all isolates demonstrated a predominance of serogroup D (*n* = 49), followed by serogroup B (*n* = 40) and serogroup C (*n* = 32) (Table 1). Among the serogroup D isolates, 65 were further evaluated using the BD Difco *Salmonella* Vi Antiserum assay, which identified 29 *S.* Typhi and 36 non-Typhi isolates. For IRBT analysis, all 164 isolates were tested following growth on Columbia agar with 5% sheep blood, while a subset of 81 isolates was additionally tested after growth on Hektoen Enteric agar. A total of 1,925 spectra were used to evaluate the performance of the *Salmonella* serogroup classifier. In addition, 790 spectra were used to assess a secondary classifier designed to discriminate between *S.* Typhi and NTS isolates within the O:9 serogroup.

**TABLE 1:** Results of the IR Biotyper (IRBT) *Salmonella* O groups classifier in comparison to Wellcolex serotyping (95% CI calculated by Clopper-Pearson method).

Wellcolex result	IRBT *Salmonella* O groups classifier results									IRBT accuracy (95% CI)	Specificity % (95% CI)	PPV % (95% CI)	NPV % (95% CI)
	O:2 (A)	O:4 (B)	O:7 (C1-4)	O:8 (C2-3)	O:9 (D1)	O:3,10 (E1-3)	O:13 (G)	Unclassified	Total				
^^Group A	4	–	–	–	–	–	–	–	4	100% (39.8–100)	96.3 (91.9–98.7)	40.0 (12.2–73.8)	100 (97.6–100)
^^Group A+E or G	5	–	–	–	–	–	–	–	5	100% (47.8–100)	100 (97.7–100)	100 (47.8–100)	100 (97.7–100)
Group B	–	40	–	–	–	–	–	–	40	100% (91.2–100)	100 (97.1–100)	100 (91.2–100)	100 (97.1–100)
Group C	1**	–	20	11	–	–	–	–	32	97% (83.8–99.9)	100 (97.2–100)	100 (88.8–100)	99.3 (95.9–100)
Group C+D	–	–	–	–	13	–	–	–	13	100% (75.3-100)	100 (97.6-100)	100 (75.3-100)	100 (97.6-100)
Group D	–	–	–	–	49	–	–	–	49	100% (92.7–100)	99.1 (95.3–100)	98.0 (89.4–100)	100 (96.8–100)
Group E or G	–	–	–	1^	1*	9	7	1*	19	89.5% (66.9–98.7)	100 (97.7–100)	100 (79.4–100)	98.0 (94.2–99.6)
^^Group Vi antigen	–	–	–	–	2	–	–	–	2	100% (15.8–100)	100 (97.7–100)	100 (15.8–100)	100 (97.7–100)
Total	10	40	20	12	65	9	7	1	164	98.2% (94.7–99.6)	Cohen’s Kappa: 0.97		

*Typed as Group E by the reference laboratory (Wellcolex results considered correct).

**Not typable by the reference laboratory (Wellcolex results considered correct).

^Typed as Group C by the reference laboratory (IRBT results considered correct).

^^The metrics reported for these groups are based on very small sample sizes and should be interpreted as caution as preliminary data.

### O-antigen classifier

Using the Wellcolex™ Color *Salmonella* kit as the reference method, the IRBT *Salmonella* O-group classifier achieved an overall accuracy of 98.2% (161/164; 95% CI: 94.7%–99.6%). To assess the reliability of the IRBT relative to conventional serotyping, Cohen’s kappa coefficient was calculated. The analysis revealed an overall kappa of 0.97, indicating almost perfect agreement between the IRBT and the Wellcolex™ Color *Salmonella* kit.

The classifier demonstrated excellent diagnostic performance across the major clinical serogroups. For Group B (*n* = 40) and Group D (*n* = 49), the system achieved a sensitivity of 100% (95% CI: 91.2%–100% and 92.7%–100%, respectively) and a specificity of 100% (95% CI: 97.1%–100% and 96.8%–100%, respectively). Detailed per-class metrics, including sensitivity, specificity, positive predictive value and negative predictive value with their corresponding 95% confidence intervals, are presented in Table 1.

While the IRBT correctly identified all isolates in Group A (*n* = 4), Group A+E or G (*n* = 5) and those expressing the Vi antigen (*n* = 2), the 100% accuracy reported for these specific subgroups is preliminary. The extremely small denominators resulted in wide 95% confidence intervals (sensitivity 95% CI for Groups A: 39.8%–100%; Vi antigen: 15.8%–100%), indicating that these point estimates are constrained by sample size.

Two isolates were misclassified: one Group C isolate was assigned to O:2 (A), and one isolate from Groups E or G was classified O:9 (D1). The IRBT failed to classify only one Groups E or G isolate. Specifically, for the 19 isolates that the Wellcolex™ Color *Salmonella* kit categorized collectively as Group E or G, the IRBT provided a more granular classification, successfully differentiating 16 of the 19 isolates (84.2%) into specific subgroups: nine isolates were identified as O:3, 10 (E1-E3) and 7 as O: 13 (G).

**FIGURE 1:**
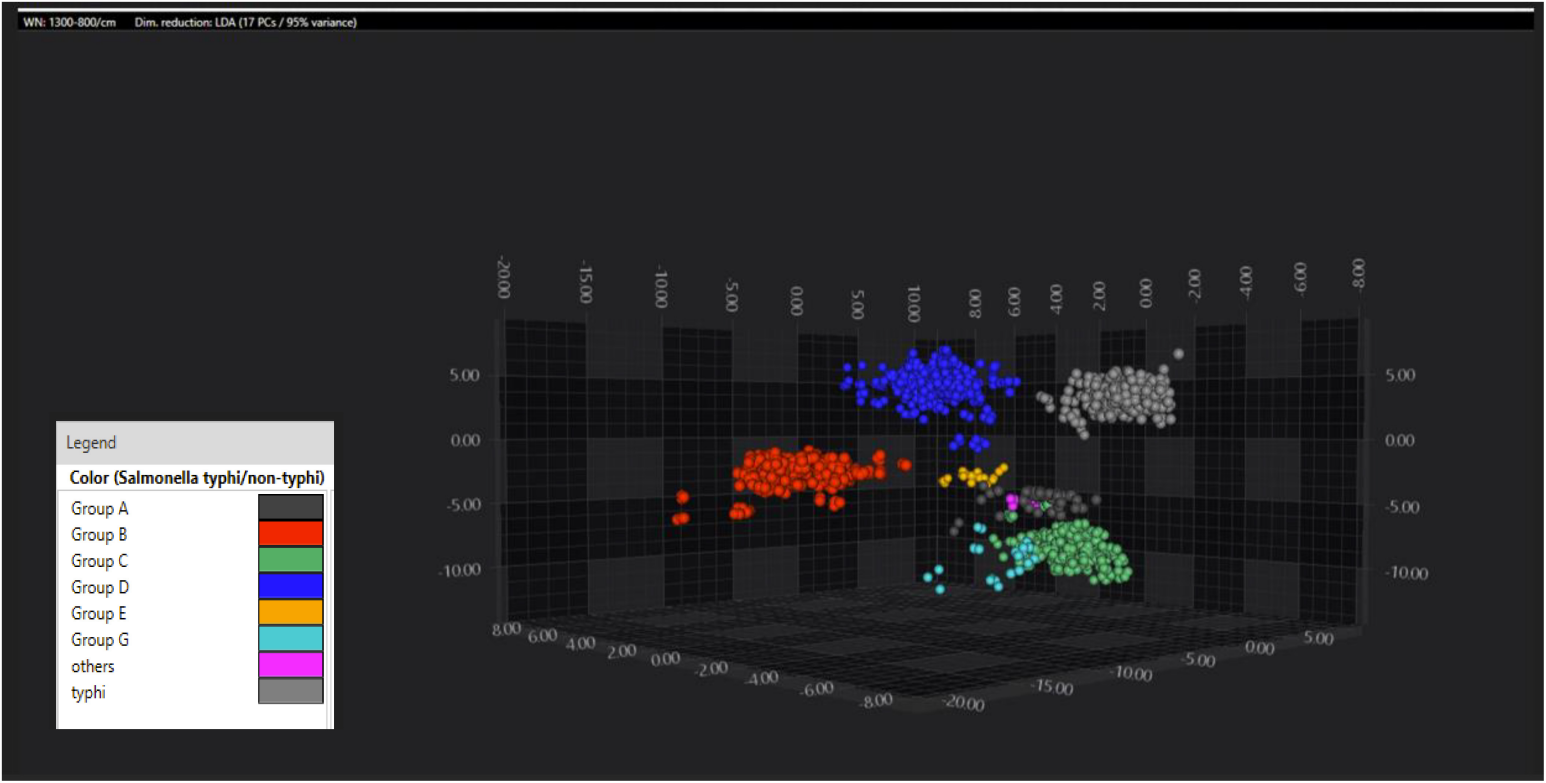
3D linear discriminant analysis (LDA) scatter plot of *Salmonella* O-serogroups in IR spectral space, showing the first three linear discriminant axes. Spectra are color-coded by O-serogroup.

Principal components analysis and linear discriminant (LDA) analysis demonstrated clear clustering of the major serogroups. PCA provided unsupervised visualization of variance-driven groupings, while LDA enhanced class separation. Most serogroups were well differentiated, indicating that infrared spectral data captured the underlying structure of the dataset. The 3D LDA scatter plot (Figure 1), based on the carbohydrates region (1,300–800 cm^–1^), was constructed using the first 20 principal components, with the first three linear discriminant axes displayed. Each point represents an individual spectrum, color-coded by O-serogroup. Distinct separation was observed for major serogroups, particularly Group B (O:4) and Group D (O:9), consistent with their 100% classification accuracy (Table 1). In contrast, partial overlap between clusters—particularly for Groups E and G—corresponded to lower classification accuracy (89.5%). To resolve these ambiguities and confirm the gold standard truth for accuracy calculations, four isolates (2.4%) required reference laboratory confirmation. The discrepancy resolution outcomes were as follows: One isolate identified as Group C by Wellcolex™ but assigned to O:2 (A) by the IRBT was found to be “not typable” by the reference laboratory; the Wellcolex™ result was treated as the truth for analysis purposes. Three isolates from the E/G group required referral. One isolate was typed as Group C by the reference laboratory, confirming the IRBT’s assignment to O:8 (C2-3) as the correct result. The remaining two isolates (one misclassified by IRBT as O:9 and one unclassified) were confirmed as Group E by the reference laboratory, supporting the initial Wellcolex™ classification. These outcomes confirmed an overall IRBT accuracy of 98.2%, with reference laboratory results serving as the definitive benchmark for resolving all discordant cases.

### Typhi vs. NTS classifier

**FIGURE 2:**
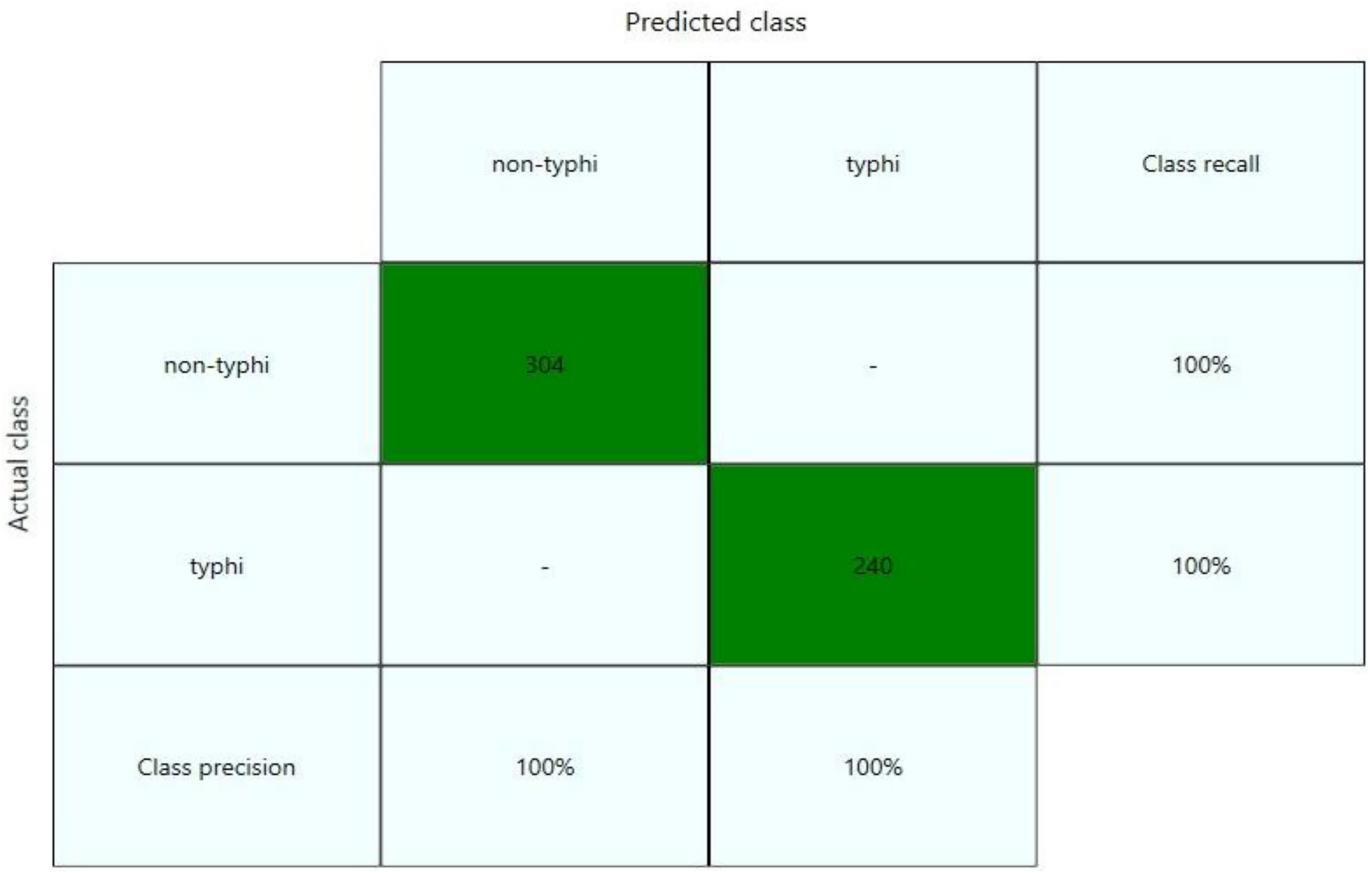
Confusion matrix for the training of Typhi vs. non-Typhi *Salmonella* (NTS) classifier. The confusion matrix is computed at the spectrum level (*n* = 544 spectra from 34 isolates).

**FIGURE 3:**
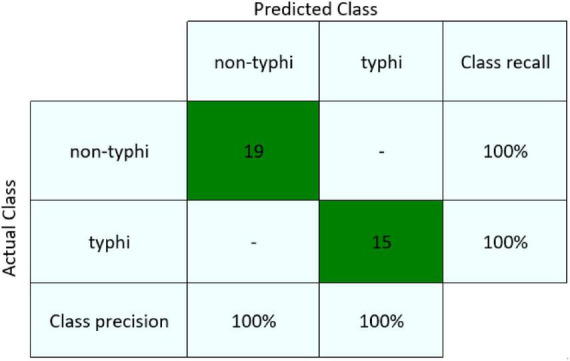
Confusion matrix for the training of Typhi vs. non-Typhi *Salmonella* (NTS) classifier. The confusion matrix is computed at the isolate level (*n* = 34).

**FIGURE 4:**
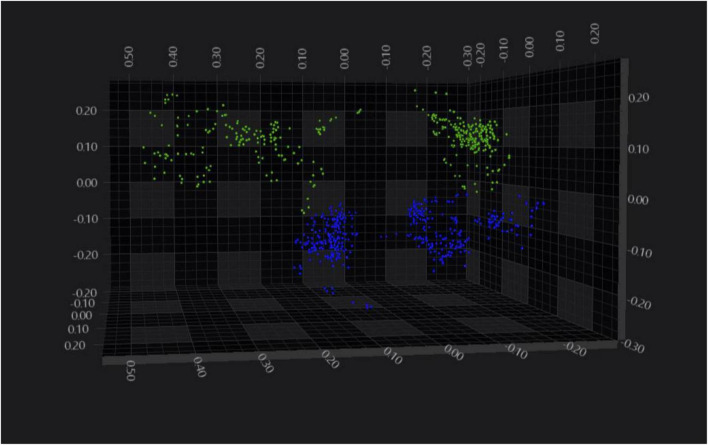
3D principal components analysis (PCA) scatter plot of *Salmonella Typhi* and non-Typhi isolates in IR spectral space, showing the first three linear discriminant axes. Spectra are color-coded by classifier output: Typhi (blue) and non-Typhi (green).

The classifier demonstrated excellent discriminatory performance in both evaluation phases. During internal cross-validation (*n* = 34 isolates; 544 spectra), the model achieved 100% accuracy (95% CI: 89.8%–100%) (Figures 2, 3). In the subsequent independent testing phase (*n* = 31 isolates; 246 spectra), the classifier correctly identified 100% of the isolates (95% CI: 88.8%–100%), demonstrating complete concordance with the reference BD Difco Salmonella Vi antiserum assay. Classifier performance is further illustrated in the 3D scatter plot (Figure 4), which shows the distribution of isolates in infrared spectral space. The visualization was generated using a PCA model based on 30 principal components, with the first three linear discriminant axes displayed. Two distinct and well-separated clusters are observed, corresponding to *S.* Typhi (blue) and NTS (green). This clear spatial separation is consistent with high classification accuracy.

## Discussion

*Salmonella enterica* is a common foodborne pathogen encountered in clinical microbiology laboratories. In this study, we evaluated the IRBT system with automated classifiers for rapid Salmonella serogrouping and *S.* Typhi differentiation, comparing its performance with conventional serotyping. This approach offers a rapid and reliable alternative to traditional methods, which are often resource-intensive and time-consuming (Fredes-García et al., 2025). By leveraging Fourier-transform infrared spectroscopy, it could significantly reduce turnaround time, enabling timely treatment decisions, improved outbreak management, and enhanced infection control measures (Gonçalves et al., 2025; Muchaamba and Stephan, 2024).

In the first part of this study, the serogroup classifier demonstrated high overall accuracy (98.2%) and strong inter-method agreement with the reference kit (κ = 0.97). Performance was optimal for major clinical serogroups, including Group B (O:4) (*n* = 40) and Group D (O:9) (*n* = 49), both achieving 100% sensitivity and specificity. While the IRBT system achieved 100% accuracy for several smaller cohorts, including Group A (*n* = 4), Group A+E or G (*n* = 5), and those expressing the Vi antigen (*n* = 2), these findings are constrained by extremely small denominators. According to standard clinical microbiology verification guidelines, a minimum of 10–50 isolates per group is typically required to provide a reliable assessment of diagnostic performance. Consequently, the 100% accuracy reported for these specific serogroups should be viewed as preliminary that necessitates further validation with larger, more diverse isolate collections to confirm the system’s discriminatory power. The observed accuracy on the serogroup classifier matches or exceeds that reported in recent FTIR-based *Salmonella* typing studies. Notably, one evaluation using ATR-FTIR demonstrated overall accuracies exceeding 98% for serogroup identification and for distinguishing between typhoidal and non-typhoidal strains (Napoleoni et al., 2024), while another study reported 100% sensitivity for serogroups O:4 and O:9 (Dopuğ et al., 2025). Accurate identification of all Group D isolates (*n* = 49), including those expressing the Vi antigen, is particularly important given the clinical relevance of typhoidal *Salmonella*. These findings are consistent with previous reports of >99% accuracy for FTIR-based classification of (para-) typhoidal *Salmonella* (Cordovana et al., 2021). Lower accuracy was observed for Groups E and G (89.5%), suggesting partial spectral overlap. This was supported by the LDA 3D scatter plot, where these groups showed close spatial proximity. Despite these limitations, the model effectively captured the underlying spectral variance, providing an objective classification framework that reduces the subjectivity associated with manual agglutination. Overall, as shown in previous studies integration of FTIR spectroscopy with machine learning demonstrates strong potential for accurate classification of diverse *Salmonella* serogroups (Campos et al., 2018; Cordovana et al., 2021, 2022; Dopuğ et al., 2025; Fredes-García et al., 2025; Napoleoni et al., 2024).

In the second part of the study, we evaluated a secondary classifier designed to discriminate between *S.* Typhi and NTS isolates within the O:9 serogroup. The classifier demonstrated excellent performance, achieving 100% accuracy in both internal cross-validation (*n* = 34) and independent testing (*n* = 31), and results were fully concordant with BD Difco Vi antiserum test. This high level of accuracy is critical for clinical diagnostics and epidemiological surveillance. Mechanistic studies have shown that virulence factors such as TolC are required for Typhi to adhere to and invade host cells and to survive intracellularly while modulating the host immune response (Hussain et al., 2023, 2024). Because Typhi resides and replicates inside host cells, where it is partially shielded from the host immune response, earlier serogroup identification permits earlier initiation of targeted intracellular-active therapy – a window in which rapid platforms such as the IR Biotyper offer a clear advantage over conventional agglutination-based workflows. Visualization of the model revealed distinct clustering patterns influenced by both bacterial type and growth medium. Specifically, spectra from isolates grown on Columbia agar with 5% sheep blood and Hektoen Enteric agar formed separate subclusters, reflecting media-dependent biochemical variation (Muchaamba and Stephan, 2024). Despite these differences, *S.* Typhi and NTS isolates remained clearly separated along the discriminant axes, indicating that the classifier captures robust, core spectral features independent of culture conditions (Muchaamba and Stephan, 2024). These findings are consistent with previous studies demonstrating > 99% accuracy for typhoidal serovars using machine learning–enhanced FTIR spectroscopy (Cordovana et al., 2021) and support the use of this approach as a rapid, reliable alternative to conventional serological methods such as the BD Difco Vi antiserum test.

The IRBT has several advantages overall traditional methods currently used in the clinical microbiology laboratories for *Salmonella* serotyping. The testing workflow is simple with minimal steps for samples preparation and short handling time. The samples can be analyzed as soon as the colonies are grown in standard media with results available within 2 h. This expediency significantly outperforms traditional serological agglutination methods, which are often time-consuming and technically complex, hindering their widespread adoption in routine diagnostics (Ren et al., 2025). Moreover, the capability of FTIR spectroscopy to offer high-throughput analysis makes it particularly suitable for large-scale epidemiological investigations and rapid identification of outbreak-related strains (Muchaamba and Stephan, 2024; Muchaamba et al., 2026). This approach, coupled with advanced machine learning algorithms, can classify various serotypes with high accuracy, offering a robust alternative to conventional methods for identifying diverse *Salmonella* serovars (Fredes-García et al., 2025; Napoleoni et al., 2024). Also, this method facilitates rapid identification of both typhoidal and NTS, crucial for timely clinical intervention and epidemiological surveillance, as demonstrated in studies differentiating key serovars within O:2, O:4, O:7, and O:9 serogroups (Cordovana et al., 2021). While the Wellcolex kit is limited by its inability to discriminate between certain serogroups (e.g., merging Groups E and G), the IRBT system utilizes a comprehensive biochemical fingerprint to provide more nuanced classification. As noted in our results, the IRBT was able to differentiate between O:13 (G) (*n* = 7) and O:3 10 (E1-E2-E3) (*n* = 9) within the 19 isolates which traditional agglutination-based kits often group together. This increased resolution is likely due to the IRBT’s ability to detect subtle variations in different cellular components within the spectral region which is often the most informative for the microbial fingerprint (1,300–800 cm^–1^), rather than relying solely on specific surface antigen-antibody reactions.

In addition, the IRBT system offers significant economic advantages in long-term clinical operations. While the initial capital investment for FTIR instrumentation can be substantial, the ongoing operational costs are remarkably low, estimated at approximately $1 per spot. In this system, only Infrared test standards are needed for quality control prior to spectra acquisition in each run. This technique has a low cost, and it only requires minimal consumables like ethanol and deionized water, contrasting sharply with the higher cost of the Wellcolex™ Color Salmonella kit, which averages about $10 per test.

It is important to acknowledge that while the IR Biotyper system demonstrates high accuracy for O-serogroup classification, it does not currently provide the full serotype identification required by the complete Kauffmann-White scheme. The KW scheme relies on the determination of both somatic (O) and flagellar (H) antigens. However, FTIR-based differentiation in *Salmonella* is primarily driven by the biochemical fingerprint of the cell surface, particularly the high carbohydrate diversity of the somatic O-antigen units (Cordovana et al., 2022). While this allows for the reliable discrimination of major serogroups such as B, C, D, and E, the system can face challenges in distinguishing between different serovars within the same O-group (Cordovana et al., 2022). Consequently, the IR Biotyper currently serves as a rapid and effective screening tool for O-grouping and the identification of high-priority strains like S. *Typhi*, but it may require supplementation with traditional serology or molecular methods for definitive flagellar antigen characterization (Cordovana et al., 2021, 2022).

Despite the high accuracy reported, several limitations of this study must be acknowledged. First, the research was designed as a single-center study conducted at a pediatric tertiary care hospital, which may restrict the generalizability of the findings to adult populations or different clinical environments. Second, the per-group sample sizes for several *Salmonella* O-groups were relatively small, notably for Group A (*n* = 4) and isolates expressing the Vi antigen (*n* = 2) which may limit the statistical robustness of the machine learning model for these specific categories. Furthermore, while the differentiation of *S.* Typhi from NTS achieved excellent results, this clinically critical sub-task was evaluated using a cohort of only 29 *S.* Typhi isolates; for context, similar studies developing automated classifiers for typhoidal serovars have utilized comparable sample sizes to achieve high accuracy, but larger, more diverse datasets are generally required for broader clinical validation (Cordovana et al., 2021).

A significant limitation is the use of the Wellcolex™ Color Salmonella kit as the primary reference standard. While widely used for rapid screening, this latex agglutination-based method has recognized limitations in sensitivity and specificity. Although we utilized reference laboratory testing to resolve discrepancies, the inherent limitations of the initial screening method may have influenced the baseline classification.

Moreover, the study lacked an external validation cohort; all independent testing was performed on isolates collected within the same institution, which may not account for the temporal diversity of *Salmonella* strains found in other regions. The evaluation was also conducted without formal operator blinding, which could introduce potential bias in sample preparation or interpretation. Furthermore, while cost-effectiveness and rapid turnaround time (under two hours) are highlighted as significant advantages of FTIR spectroscopy, we did not perform a formal, head-to-head cost analysis or a time to result comparison with traditional methods. Such a comparison would be necessary to quantify the exact economic and operational benefits in a high-volume clinical workflow (Gonçalves et al., 2025; Muchaamba and Stephan, 2024).

An additional limitation of this study is the lack of a head-to-head comparison with MALDI-TOF MS combined with machine learning, a parallel advancement in rapid *Salmonella* typing. However, while MALDI-TOF MS is used at our institution for routine genus-level identification, its application for serotyping currently requires specialized bioinformatic pipelines and external software to interpret mass spectra, which are not yet integrated into standard clinical workflows (Ren et al., 2025). In contrast, the IR Biotyper system provides an automated, turn-key solution for O-serogroup classification by analyzing the high antigenic diversity of surface structures (Cordovana et al., 2022; Napoleoni et al., 2024). Future research should prioritize comparative studies between these two phenotypic methods to evaluate their respective diagnostic sensitivities and ease of integration into clinical settings.

Another limitation of this study pertains to the statistical precision of performance metrics for O-serogroups with small denominators. While the IR Biotyper achieved high accuracy for Group A (*n* = 4), Group A+E or G (*n* = 5), and the Vi antigen (*n* = 2), these point estimates are associated with wide 95% confidence intervals, reflecting high statistical uncertainty. In clinical diagnostic evaluations, such small sample sizes are insufficient to establish definitive sensitivity, specificity, or predictive values (PPV/NPV). Consequently, these results should be interpreted as preliminary indicators of the system’s potential rather than conclusive evidence of its diagnostic precision for these rare serotypes. Despite these constraints, the high level of agreement (κ = 0.97) between the IRBT and the reference method suggests that the system is a robust tool for rapid screening.

Despite its advantages, the application of FTIR spectroscopy for *Salmonella* typing remains limited by the need for comprehensive spectral databases that capture the diversity of *S. enterica* serovars, as well as variability introduced by differences in growth conditions and sample preparation (Muchaamba and Stephan, 2024). Standardization is therefore essential to ensure reproducibility across laboratories and platforms (Novais et al., 2024). Nonetheless, previous studies have demonstrated that FTIR can produce reproducible results across varying conditions and instruments (Rodrigues et al., 2020). Further improvements in analysis algorithms and expansion of validated spectral libraries will be important to enhance discrimination, particularly for closely related serovars, and to support broader clinical implementation.

In conclusion, our study demonstrates that the IRBT system is a promising, user-friendly tool for *Salmonella* typing at the O-serogroup level and for differentiating *S.* Typhi from NTS isolates. This approach offers a substantial improvement over conventional methods by reducing the time and resources required for accurate serovar identification, with potential benefits for public health response to outbreaks. Further studies are needed to enhance the robustness of this method by incorporating a broader range of *Salmonella* serogroups and increasing spectral diversity across different culture media. In addition, integration of FTIR spectroscopy with other advanced analytical approaches may further improve diagnostic accuracy and support the development of comprehensive platforms for rapid *Salmonella* detection and serovar identification.

## Data Availability

The raw data supporting the conclusions of this article will be made available by the authors, without undue reservation.
